# Supplementary material for: Bacterial Lysates Modulate Human Macrophage Responses by Inducing BPI Production and Autophagy
Source: Biomolecules. 2025 Oct 13;15(10):1446. doi: 10.3390/biom15101446 (PMC12562339; doi:10.3390/biom15101446)
Supplement: Supplementary file 1 [file biomolecules-15-01446-s001.zip › biomolecules-3864376-Original Western blot.pdf]

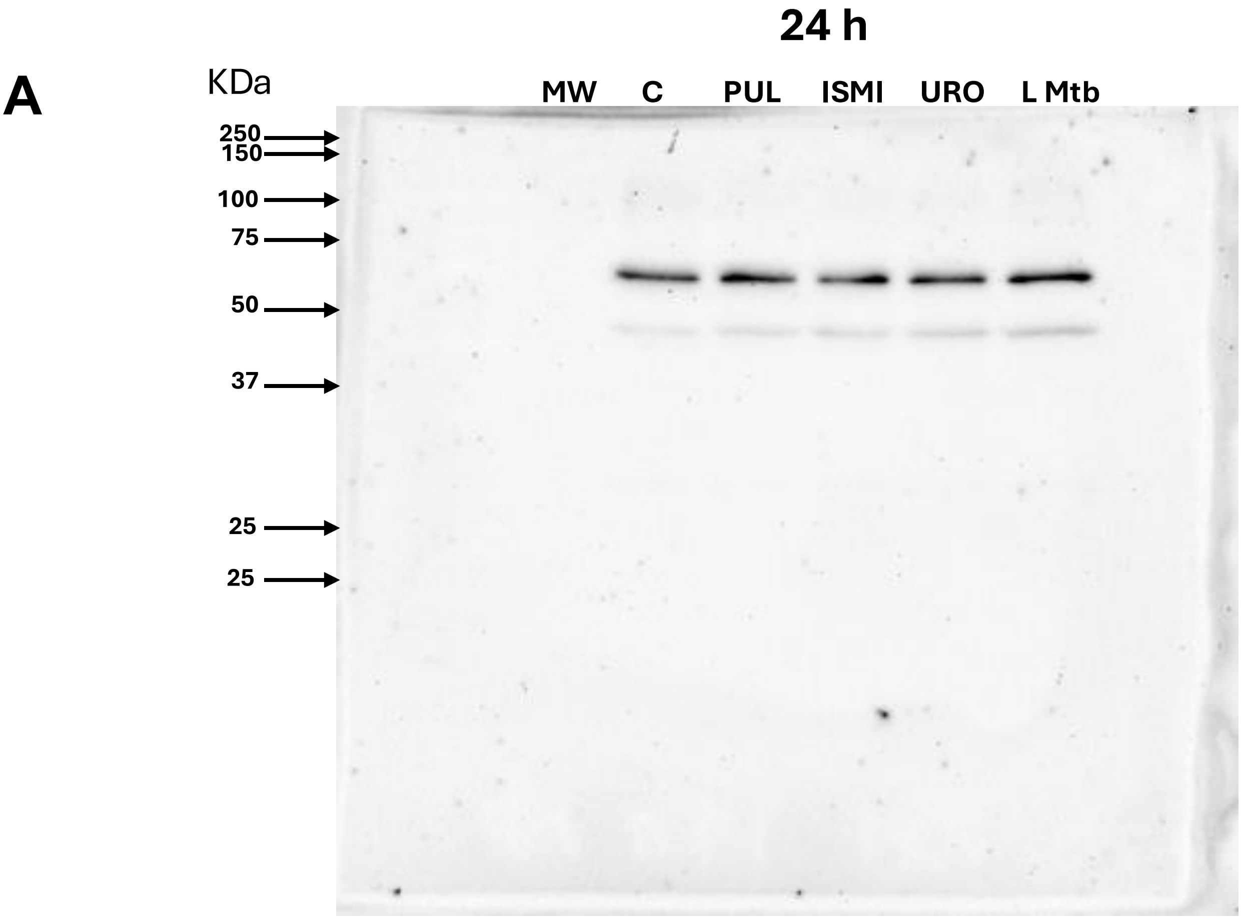

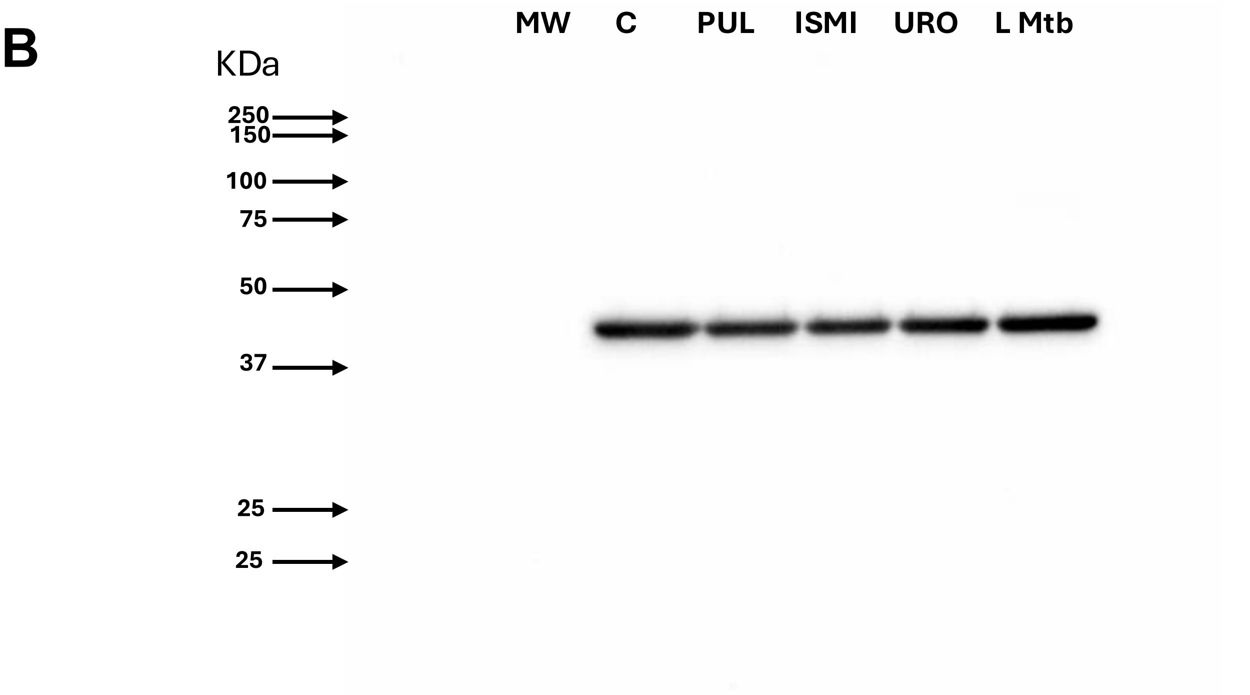

Original Western blot showing (A) human BPI protein expression and (B) β-actin (loading control) in macrophages stimulated for 24 h with bacterial lysates. Lane order: molecular weight marker (MW), control (C), Pulmonarom (PUL), Ismigen (ISM), Uro-Vaxom (URO), *M. tuberculosis* lysate (LMtb).

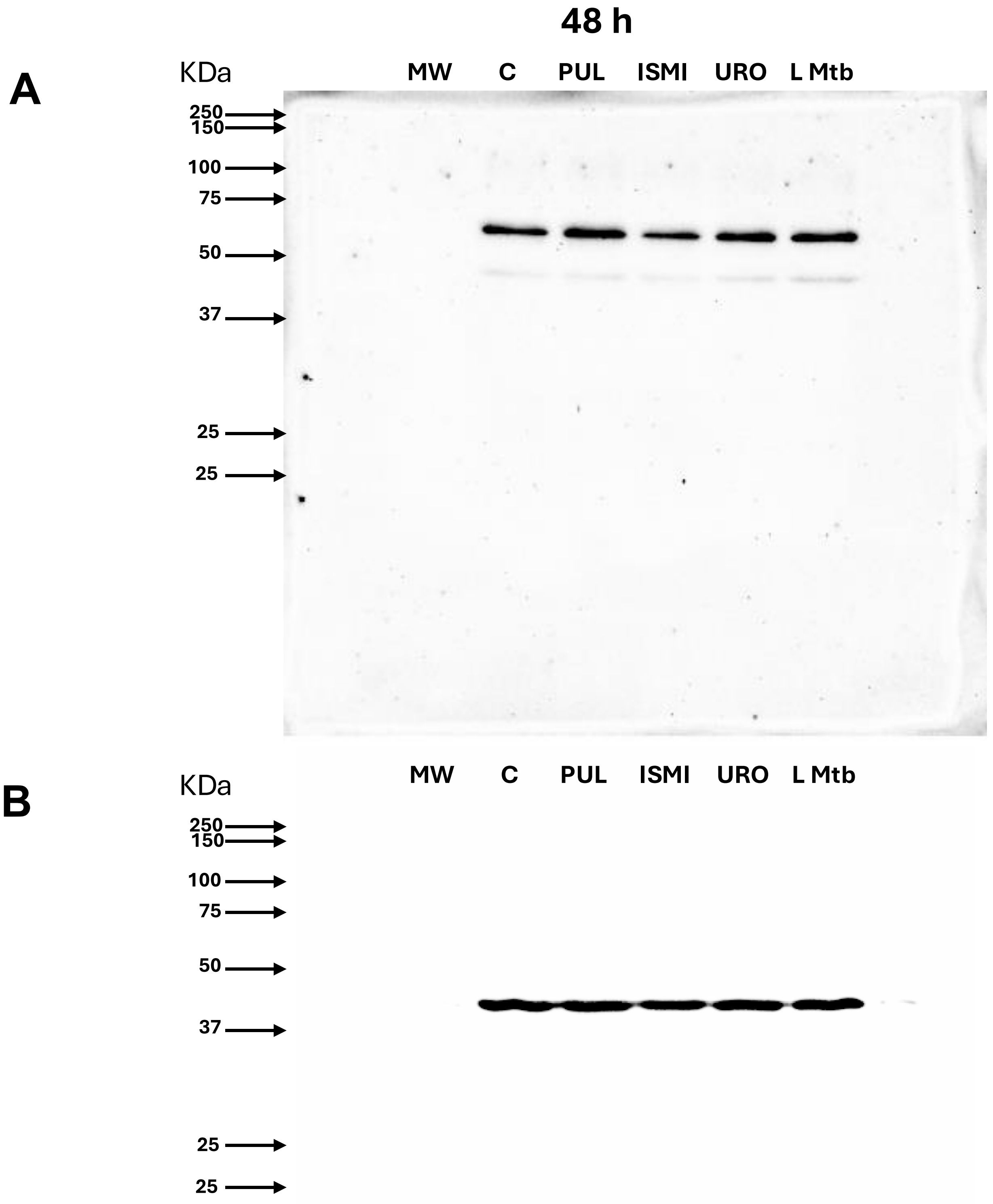

Original Western blot showing (A) human BPI protein expression and (B) β-actin (loading control) in macrophages stimulated for 48 h with bacterial lysates. Lane order: molecular weight marker (MW), control (C), Pulmonarom (PUL), Ismigen (ISM), Uro-Vaxom (URO), *M. tuberculosis* lysate (LMtb).

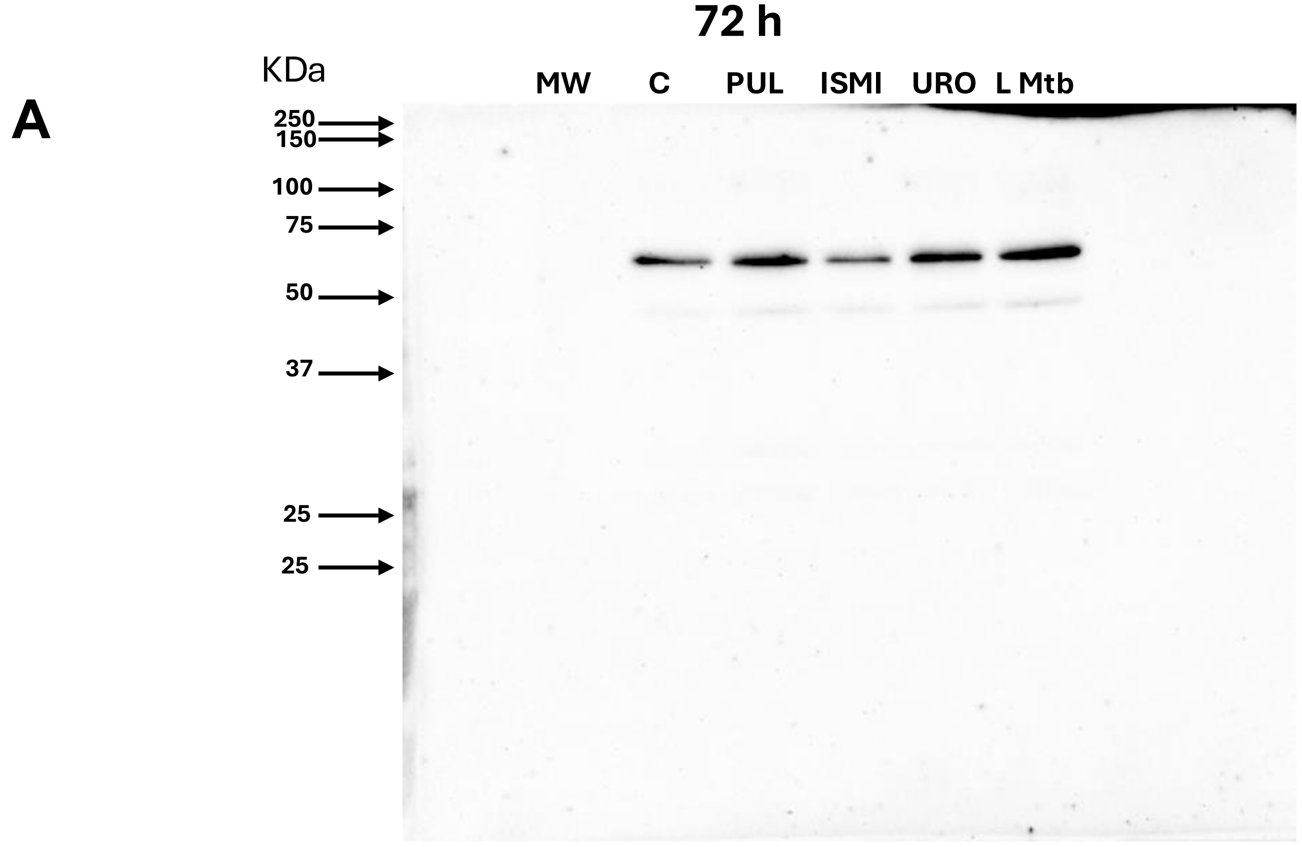

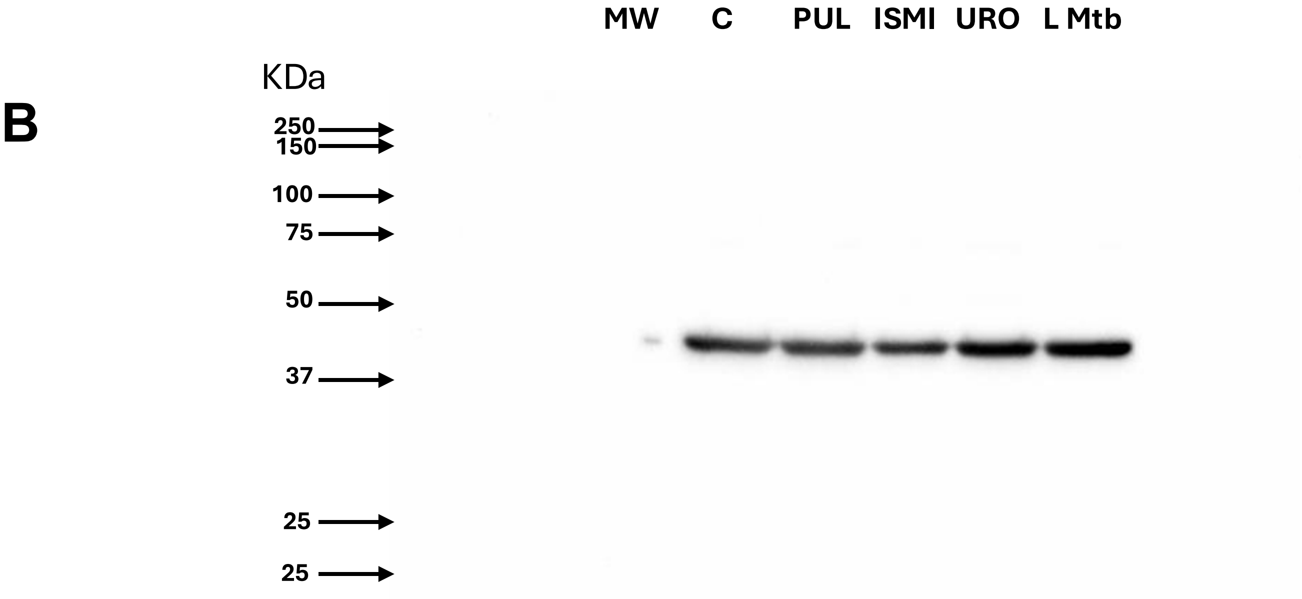

Original Western blot showing (A) human BPI protein expression and (B) β-actin (loading control) in macrophages stimulated for 72 h with bacterial lysates. Lane order: molecular weight marker (MW), control (C), Pulmonarom (PUL), Ismigen (ISM), Uro-Vaxom (URO), *M. tuberculosis* lysate (LMtb).
