# Supplementary material for: Bacterial Lysates Modulate Human Macrophage Responses by Inducing BPI Production and Autophagy
Source: Biomolecules. 2025 Oct 13;15(10):1446. doi: 10.3390/biom15101446 (PMC12562339; doi:10.3390/biom15101446)
Supplement: Supplementary file 1 [file biomolecules-15-01446-s001.zip › Supplementary Figures v3.pdf]

Supplementary Table S1. Bacterial lysate composition

	PULMONAROM [1,2]	ISMIGEN [3,4]	URO VAXOM [5]
	Haemophilus influenzae 6 x 10^8 CFU<br>*Staphylococcus aureus* 6 x 10^8 CFU<br>*Moraxella catarrhalis* 6 x 10^8 CFU<br>*Klebsiella pneumoniae* 6 x 10^8 CFU<br>*Streptococcus pneumoniae* 6 x 10^8 CFU<br>*Streptococcus pyogenes* 6 x 10^8 CFU<br>*Streptococcus agalactiae* 6 x 10^8 CFU<br>*Streptococcus dysgalactiae* 6 x 10^8 CFU<br>*Streptococcus anginosus* 6 x 10^8 CFU<br><br>Vehicle cbp 3 mL	*Staphylococcus aureus* 6.0 x 10^9 CFU<br>*Streptococcus pyogenes* 6.0 x 10^9 CFU<br>*Streptococcus viridans* 6.0 x 10^9 U CFU<br>*Klebsiella pneumoniae* 6.0 x 10^9 CFU<br>*Klebsiella ozaenae* 6.0 x 10^9 CFU<br>*Haemophilus influenzae* tipo B 6.0 x 10^9 U CFU<br>*Moraxella (Neisseria) catarrhalis* 6.0 x 10^9 CFU<br>*Streptococcus pneumoniae (Diplococcus)* 6.0 x 10^9 CFU<br>Include (TY1/EQ11, TY2/EQ22, TY3/EQ14, TY5/EQ15, TY8/EQ23 y TY47/EQ24)	18 lyophilized *Escherichia coli* strains used in recurrent urinary tract infections
Formulation and administration	Oral/Ampoules (contain 60 x 10^6 UFC/ 3ml)	50 mg bacterial lysate per capsule Oral	6 mg bacterial lysate per capsule Oral
Preparation method	Mechanical lysis Homogenization, sonication, agitation with glass beads,	Mechanical lysis Physical disruption via pressure, sonication, or abrasion	Controlled chemical lysis using detergents and enzymes, followed by detoxification and lyophilization

1. Pulmonarom, bacterial lysates solution. (https://www.medicamentosplm.com/Home/productos/pulmonarom.solucion/162/101/9468/146).
2. Hernández-Aceves JA, Solano-Gálvez SG, Wilkins-Rodríguez AA, Delgado-Domínguez J, Lozano AG, Cabello-Gutierrez C, Huerta LFE, Fragoso G, Gutiérrez-Kobeh L, Vázquez-López R. Immunomodulatory Effects of Pulmonarom®: In Vitro Induction of TLR and Cytokine Expression in Human Dendritic Cells. Pharmaceuticals (Basel). 2025 Jun 13;18(6):885.
3. Ismigen sublingual tablets. (https://www.medicamentosplm.com/Home/productos/ismigen.tabletas/115/101/44708/210)
4. Rahman MM, Talukder A, Rahi MS, Das PK, Grice ID, Ulett GC, Wei MQ. Evaluation of Immunostimulatory Effects of Bacterial Lysate Proteins on THP-1 Macrophages: Pro-inflammatory Cytokine Response and Proteomic Profiling. J Immunol Res. 2025 Apr 25;2025:2289241.
5. Volontè S, De Vicari D, Cola A, Barba M, Frigerio M. Efficacy and Safety of Uro-Vaxom in Urinary Tract Infection Prevention: A Systematic Literature Review. J Clin Med. 2025 May 29;14(11):3836.

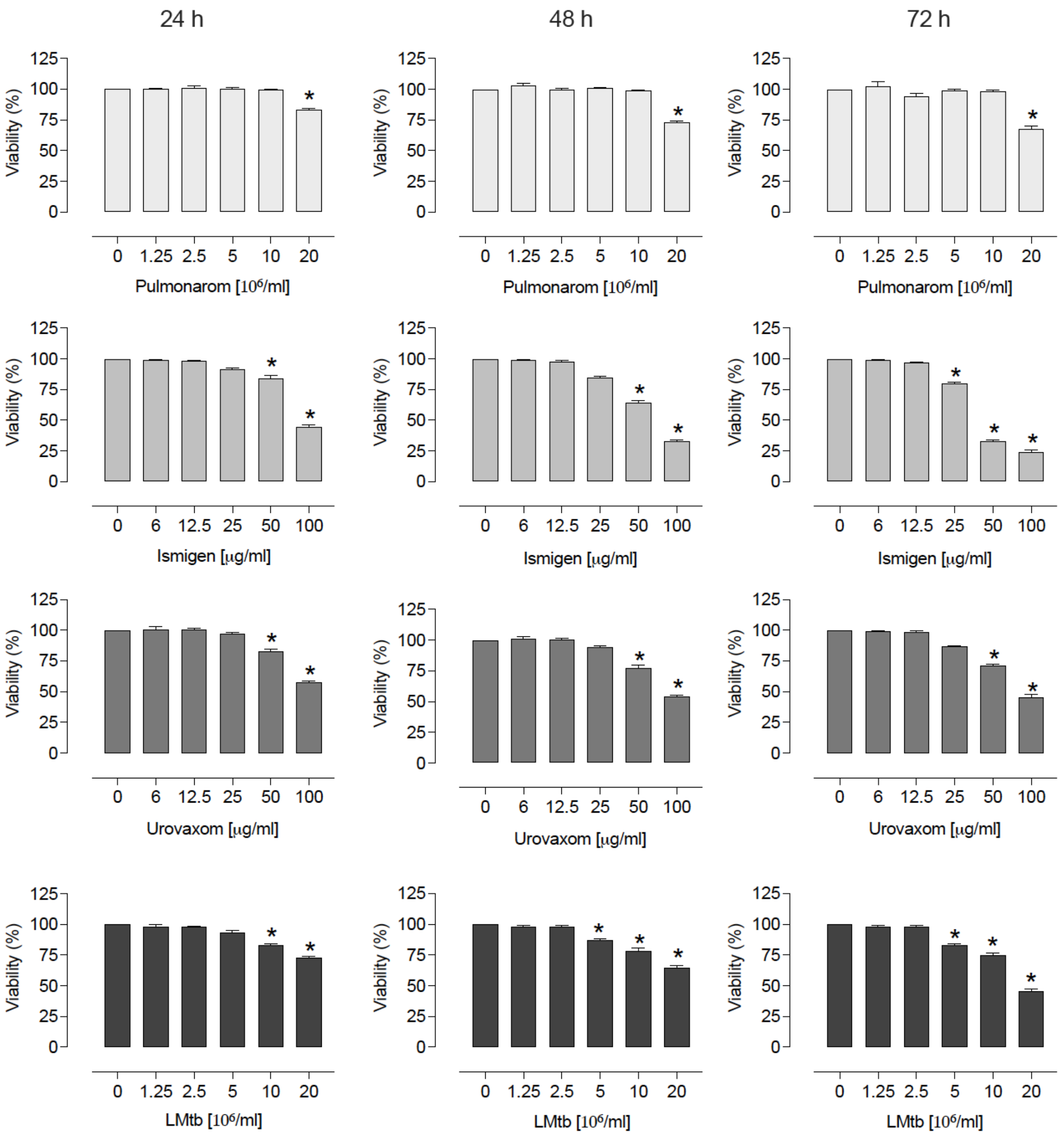

Supplementary Figure S1. Cytotoxic effect of commercial bacterial lysates on MDMs. Cells were treated with increasing concentrations of Pulmonarom (1.25–20×10^6/ml), Urovaxom, Ismigen (6–100 µg/ml), or *Mycobacterium tuberculosis* lysate (LMtb; 1.25–20×10^6/ml) for 24, 48, or 72 hours. Cell viability was determined using the MTT assay and is expressed as a percentage relative to the untreated control (set at 100%). Data represent the mean ± SD (n =6). $p < 0.05$ compared to untreated control.

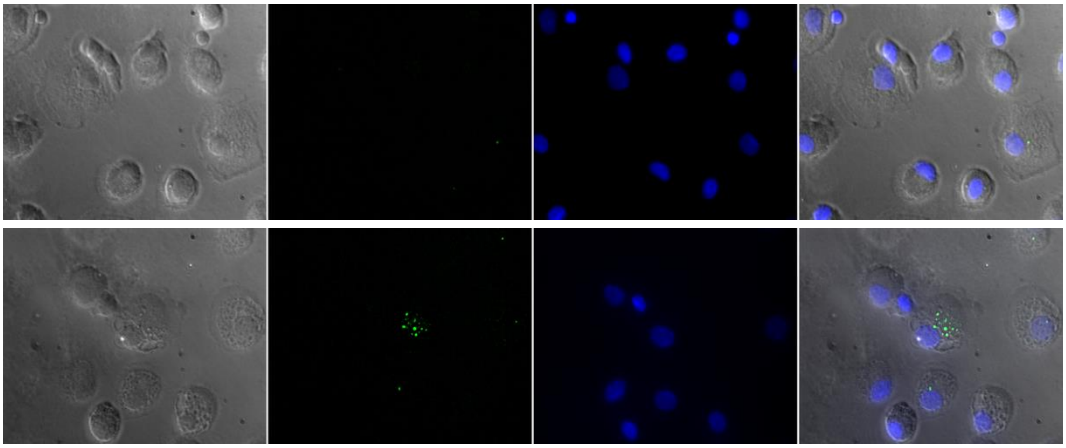

Supplementary Figure S2. Human rBPI induces autophagy in macrophages. Cells were stimulated with recombinant Bactericidal/Permeability-Increasing Protein (rBPI; 250 ng/mL) for 48 h. Autophagosomes were visualized by fluorescence microscopy using anti-human LC3-FITC. Merged images show LC3-positive cells. Representative images are shown from five independent experiments and were acquired at 100× magnification. Control cells were untreated.

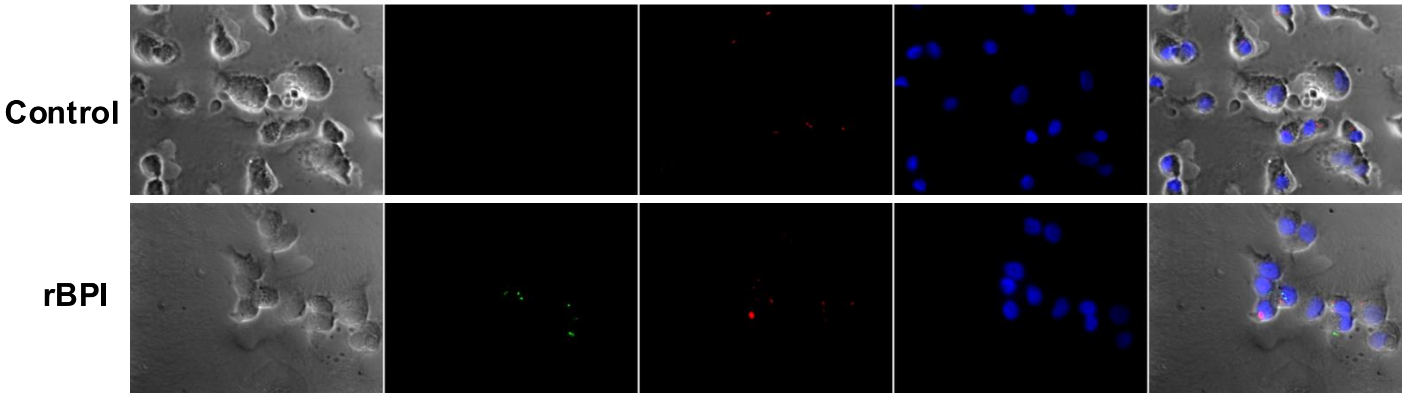

Supplementary Figure S3. Human rBPI induces autophagy in infected macrophages. Cells were stimulated with recombinant Bactericidal/Permeability-Increasing Protein (rBPI; 250 ng/mL) for 48 h and subsequently infected with *M. tuberculosis*. Autophagosomes were visualized by fluorescence microscopy using anti-human LC3-FITC. Merged images show LC3-positive cells. Representative images are shown from five independent experiments and were acquired at 100× magnification. Control cells were untreated.
